# Fueling Cancer Immunotherapy With Common Gamma Chain Cytokines

**DOI:** 10.3389/fimmu.2019.00263

**Published:** 2019-02-20

**Authors:** Connor J. Dwyer, Hannah M. Knochelmann, Aubrey S. Smith, Megan M. Wyatt, Guillermo O. Rangel Rivera, Dimitrios C. Arhontoulis, Eric Bartee, Zihai Li, Mark P. Rubinstein, Chrystal M. Paulos

**Affiliations:** ^1^Department of Microbiology and Immunology, Medical University of South Carolina, Charleston, SC, United States; ^2^Department of Dermatology and Dermatologic Surgery, Medical University of South Carolina, Charleston, SC, United States; ^3^Department of Surgery, Medical University of South Carolina, Charleston, SC, United States

**Keywords:** chimeric antigen receptor, T cell, adoptive cell transfer, gamma chain cytokines, TRUCKs

## Abstract

Adoptive T cell transfer therapy (ACT) using tumor infiltrating lymphocytes or lymphocytes redirected with antigen receptors (CAR or TCR) has revolutionized the field of cancer immunotherapy. Although CAR T cell therapy mediates robust responses in patients with hematological malignancies, this approach has been less effective for treating patients with solid tumors. Additionally, toxicities post T cell infusion highlight the need for safer ACT protocols. Current protocols traditionally expand T lymphocytes isolated from patient tumors or from peripheral blood to large magnitudes in the presence of high dose IL-2 prior to infusion. Unfortunately, this expansion protocol differentiates T cells to a full effector or terminal phenotype *in vitro*, consequently reducing their long-term survival and antitumor effectiveness *in vivo*. Post-infusion, T cells face further obstacles limiting their persistence and function within the suppressive tumor microenvironment. Therapeutic manipulation of T cells with common γ chain cytokines, which are critical growth factors for T cells, may be the key to bypass such immunological hurdles. Herein, we discuss the primary functions of the common γ chain cytokines impacting T cell survival and memory and then elaborate on how these distinct cytokines have been used to augment T cell-based cancer immunotherapy.

## Introduction

The field of cancer immunotherapy, encompassing vaccines, checkpoint modulators, and adoptive T cell transfer therapy (ACT), has improved treatment outcomes in patients by harnessing the immune system to target their malignancy, sometimes resulting in cures (1). ACT uses either tumor-infiltrating lymphocytes (TILs) already equipped with tumor-specificity or peripheral blood lymphocytes genetically redirected with tumor-specific T cell receptors (TCRs) or chimeric antigen receptors (CARs) (2). Two different groups in the 1980's first revealed that T cells could be successfully redirected with an antigen receptor. Kuwana and team engineered a CAR that combined the immunoglobulin variable regions with a TCR constant region and they reported specificity against phosphorylcholine-specific bacteria (3). Gross et al. then used a similar construct but made the transformants specific for TNP-expressing cancer cell lines. They demonstrated that these CAR T cells could secrete IL-2 and lyse tumor cells in an antigen-specific manner (4). In some instances, engineering cells with a CAR instead of a TCR can be advantageous. This advantage stems from the fact that CARs, similar to antibodies, are able to recognize free unmodified antigen while TCRs require antigen modification and presentation by the major histocompatibility complex (MHC), which is often down-regulated on tumor cells (5). However, unlike TCRs, CAR antigen specificity is restricted to cell surface antigens.

Following the two initial studies, CAR designs have been further modified to enhance their antitumor properties and persistence. First-generation CARs use a single chain variable fragment (scFv) for antigen recognition and an intracellular signaling domain, CD3ζ or FcεRIγ (6). In recent years, the incorporation of one or more co-stimulatory domains (i.e., second and third generation CARs) was instrumental to the success of CAR T cell efficacy for patients in clinical trials. As reviewed by Knochelmann et al., donor lymphocytes have been further modified in many ways by (1) incorporating targets to multiple antigens, (2) converting suppressive signals such as TGF-β or IL-4 into activating signals, (3) overexpression of chemokine receptors to enhance migration, and (4) secreting cytokines or soluble factors to modulate donor TIL or CAR T cells and endogenous immune cell function to induce a proinflammatory or “hot” tumor microenvironment (7, 8).

The most notable recent successes with CAR T cell therapy have resulted from the use of second generation CD19-CAR T cells for B cell derived malignancies that incorporate CD28 or 4-1BB costimulatory domains. Administration of CD19-CAR T cells leads to near complete eradication of CD19^+^ malignant and B cell lineage cells in patients with advanced lymphomas (9–14) and multiple forms of chemo-refractory or advanced leukemias (15–23). In many of these studies, CAR T cell therapy induced long term remissions in patients who had been heavily pre-treated with various ineffective therapies. Due to their unprecedented success in multiple patients in clinical trials, the second generation CD19-CAR containing 4-1BB-CD3ζ (Tisagenlecleucel) was FDA approved for patients with B cell acute lymphoblastic leukemia in 2017 and diffuse large B cell lymphoma in 2018 while the second generation CD19-CAR containing CD28-CD3ζ (Axicabtagene ciloleucel) was approved for diffuse large B cell lymphoma in 2017 (7). Indeed, these therapies have revolutionized treatment for many patients around the world suffering from advanced hematological malignancies.

Though CAR T cell therapy has demonstrated incredible success with certain hematologic cancers, challenges still remain today in using this therapy to successfully treat patients with solid tumors. There also remains challenges in managing treatment-associated toxicity. Toxicities associated with CAR T cell therapy can be numerous including (1) cytokine release syndrome (CRS) which is characterized by a fever induced by high serum levels of IL-6 and IFNγ (2) respiratory distress and (3) neurological symptoms (23–26). All of these toxic side effects can be lethal in individuals if left untreated (23–26). To manage these adverse events, patients are treated with drugs to block CRS such as IL-6 inhibition with tocilizumab (anti-IL-6R) or corticosteroids (23, 24). For the treatment of solid tumors, CAR T cell therapies have poor efficacy due to tumor mediated suppression by (1) inhibitory receptor engagement, (2) soluble factors, (3) recruitment of suppressive immune cells, (4) nutrient deprivation and (5) loss of tumor antigen (5, 27–30). As solid tumor-specific antigens are difficult to identify, patients can experience toxic side effects due to on-target off-tumor reactivity leading to autoimmune-like symptoms (26, 31–37). Consequently, investigators have more recently designed CAR T cell constructs containing an inducible suicide gene to rapidly eliminate CAR T cells from the patient with a pharmacological reagent. The hope is that this approach will theoretically reverse or reduce the onset of these adverse events (38–42).

Novel ways to improve the potency of CAR T cells in the tumor are desperately needed for patients that fail conventional chemotherapies or other forms of cancer immunotherapy. T cell function, survival, and proliferation are strongly influenced by cytokine signaling. Notably, the members of the common γ chain (γc) cytokine family play pivotal roles in fueling T cells to thrive, lyse tumors and drive long-lived memory to tumor relapse or metastasis. While IL-2 has been widely used to expand T cells *ex vivo* in preparation for infusion into patients, preclinical work reveals that other members of the γc cytokine family should be considered for clinical use. Consequently, this review will detail the basic biology of various γc cytokines, including IL-2, IL-4, IL-7, IL-9, IL-15, and IL-21 and discuss how each cytokine has have been used in cellular therapy. Lastly, we will discuss a subset of fourth generation CARs known as TRUCKs (T cell redirected for universal cytokine-mediated killing) in cancer immunotherapy and discuss our vantage of how to best augment their antitumor potency using γc cytokines *in vitro* and *in vivo* to safely improve treatment outcomes in patients with advanced blood or solid tumors.

## Overview: Common γ Chain Cytokine Signaling and Function in T Lymphocyte Biology

**Figure 1:**
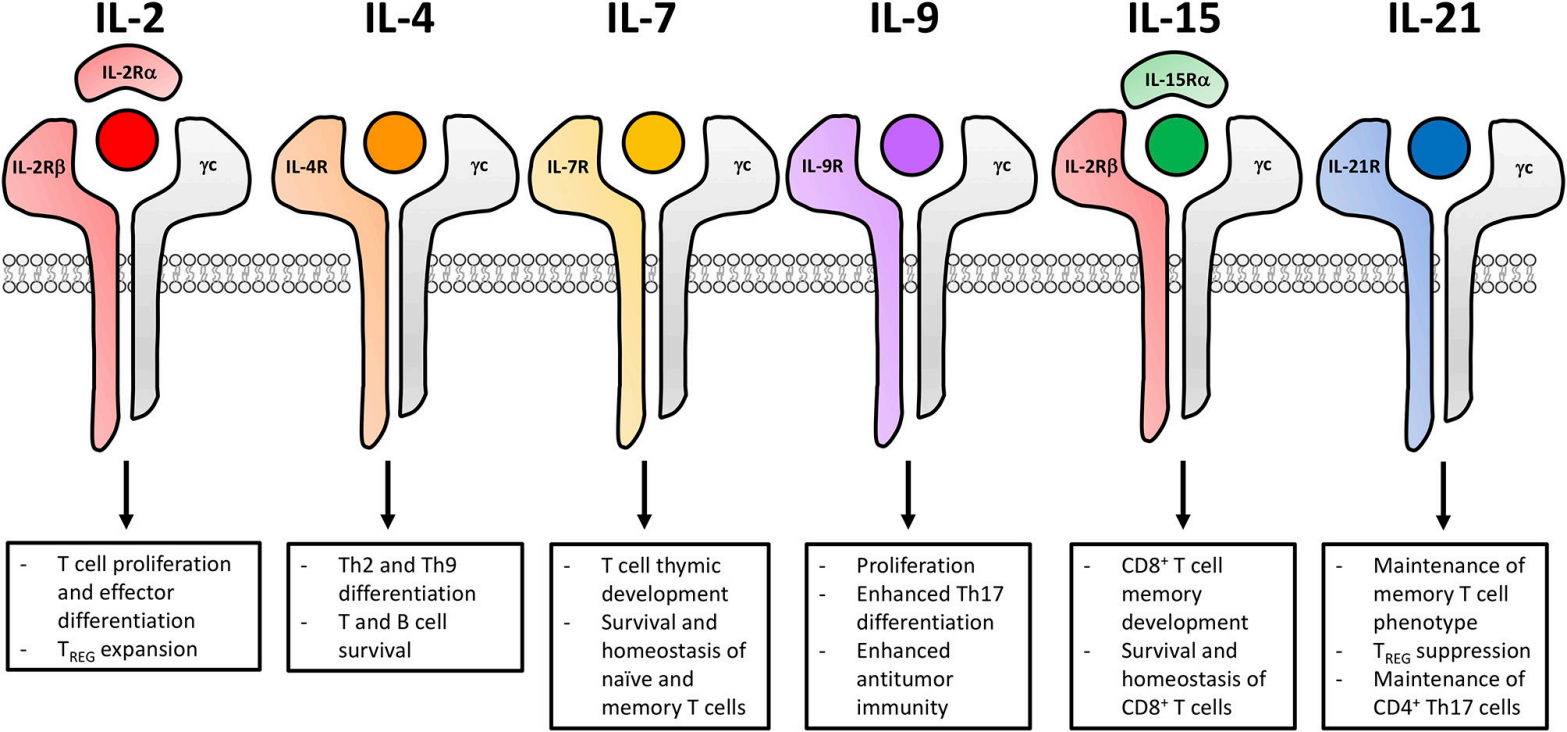
Common γ chain cytokine signaling impacts the functional fate of T cells for adoptive cell transfer. The six members of the γc cytokine family (IL-2, IL-4, IL-7, IL-9, IL-15, and IL-21) and the composition of their unique cytokine receptors. Signaling cascades from these receptors lead to distinct biological outcomes impacting differentiation, effector function and memory development of T cells.

Common γ chain cytokines exert numerous functions on T lymphocyte survival, function and proliferation. As illustrated in Figure 1, the γc family consists of six members—IL-2, IL-4, IL-7, IL-9, IL-15, and IL-21—which all have unique receptors. Upon receptor ligation, γc cytokines through JAK1 and JAK3 activate various developmental pathways including STAT1, STAT3, STAT5, MAPK, and PI3K/AKT pathways (43–55). The one exception is IL-4, which in addition to STAT5, MAPK and PI3K/AKT pathways, activates STAT6 signaling (56–62). Below, we will further discuss receptor composition and the biological functions exerted by each of these six γc cytokines.

### IL-2

IL-2 is primarily produced by activated T cells upon TCR and costimulatory signaling (43). As displayed in Figure 1, the IL-2 receptor (IL-2R) is a trimeric receptor that consists of IL-2Rα, IL-2Rβ and the γc where signaling is ultimately mediated through IL-2Rβ and the γc (43, 44). High affinity IL-2Rs (α*βγ*) are expressed on activated T cells and constitutively expressed on T regulatory cells (Tregs) while the intermediate affinity IL-2R (βγ) is expressed on natural killer (NK) cells and memory CD8^+^ T cells (43). IL-2 has non-redundant functions in both Treg and effector T cell biology. For Tregs, IL-2 is essential for thymic development, peripheral homeostasis, and suppressive function (63–74). In IL-2-, IL-2Rα-, and IL-2Rβ-deficient models, mice succumb to lethal autoimmunity within 8–12 weeks due to impaired thymic development of Tregs (63–66). Conversely, effector T cells readily develop in IL-2-, IL-2Rα-, and IL-2Rβ-deficient models. However, IL-2 is essential for the optimal proliferation and differentiation of effector T cells and this cytokine influences their contraction through activation induced cell death (AICD) (75). Additionally, IL-2 plays distinct roles in the function of various CD4^+^ T helper (Th) subsets. The differentiation of Th1, Th2, Th9, and iTreg subsets is promoted by increased expression of IL-2 while Th17 and Tfh differentiation is suppressed by IL-2 (76–81). IL-2R signaling intensity influences the development, survival and recall response of T cell memory (82–85). Low IL-2R signaling favors the development of central memory T cells (Tcm) whereas high IL-2R signaling favors the development of effector memory T cells (Tem) and terminally-differentiated effector cells (86). IL-2 also influences effector development through the upregulation of IFNγ, perforin, granzyme B and Blimp-1, which drive terminal effector differentiation and suppresses expression of makers associated with memory (such as Bcl-6, CD127, and CD62L) (86–89).

### IL-4

The cytokine IL-4 has long been appreciated to impact humoral immunity. IL-4 is primarily produced by CD4^+^ T cells (specifically Th2 and Tfh cells), basophils, eosinophils, mast cells and NKT cells (90–98). Along with the γc receptor, IL-4 binds to IL-4Rα (Figure 1). Upon IL-4 receptor (IL-4R) signaling, cascades promote the up-regulation of IL-4Rα, which induces a positive feedback loop (62, 99, 100). IL-4 is required for the differentiation of naïve CD4^+^ T cells to a Th2 phenotype. This cytokine also induces and immunoglobulin class switching in B cells, promotes the survival of T and B cells and drives long-term development of CD8^+^ T cell memory (101). Humoral immunity is dependent on IL-4, as IL-4-, or IL-4R-deficient mouse models have impaired antibody production, high susceptibility to parasitic infection and diminished Th2 differentiation (101). IL-4 is thought to be controversial for cancer therapy because multiple forms of cancer express the IL-4R. Increased IL-4R expression has been observed in renal cell carcinoma, melanoma, breast, glioblastoma, lung, prostate, bladder and head neck cancers (102–107). Angiogenesis of human breast tumor cells has been shown to be inhibited by the addition of IL-4, preventing metastatic growth and proliferation (108, 109). However, since both adipose tissue and cancer cells secrete IL-4 to promote a suppressive tumor microenvironment, blocking IL-4R signaling was found to decrease the viability of breast tumor cells (110). Finally, recent data has emerged that, along with TGF-β, IL-4 can support the generation of a new subset called Th9 cells. These cells secrete IL-9 and have been reported to augment immunity to tumors in ACT models (111, 112). Indeed, future investigations are required to better understand the role of IL-4 in regulating Th2 and Th9 cells in adoptive immunotherapy for cancer.

### IL-7

In contrast to IL-2, cytokine IL-7 is not produced by hematopoietic cells but rather is secreted by stromal cells (113–115). Its receptor consists of the γc and a unique IL-7Rα (Figure 1) (113). The fundamental role of IL-7 has been demonstrated in both humans and in mice with deficiency in either IL-7 or the IL-7 receptor (IL-7R) resulting in impaired thymic development of mature lymphocytes resembling severe combined immune deficiency (116–118). Moreover, IL-7 supports the survival and homeostasis of naïve and memory T cells (119–124). Upon activation and IL-7R signaling, the IL-7R is down-regulated on naïve T cells. Interestingly, IL-7R is re-expressed on Tcm and Tem cells (125–128). It is important that cells express IL-7R as IL-7 signaling promotes the homeostasis and survival of naïve and memory T cells via the up-regulation of Bcl-2 and the suppression of pro-apoptotic mediators (49, 129–131). Unlike IL-2, IL-7 does not induce Treg proliferation, as IL-7Rα is expressed at low levels on this suppressive lymphocyte population (132, 133). Due to the deleterious role of Tregs in cancer immunotherapy, many investigators are now exploring the role of IL-7 in potentiating checkpoint modulators or T cell therapies, as discussed in greater detail later in this review.

### IL-9

IL-9 was initially described as a T cell growth factor. However, IL-9 is more recently appreciated for its role in the proliferation and differentiation of mast cells as well as its involvement in B cell maturation (134–137). IL-9 is primarily produced by various CD4^+^ T cell subsets (naïve, Th2, Th9, Th17, and Tregs) but can also be made by mast cells, NKT cells and type 2 innate lymphoid cells (ILC2) (112, 138–145). As shown in Figure 1, IL-9 signals through the γc and IL-9Rα which is expressed on activated T cells, mast cells and macrophages (52, 146). In IL-9- and IL-9 receptor (IL-9R)-deficient mice, there was no effect on T cell differentiation or development, but these mice had diminished mast cell proliferation (147). Additional investigations revealed that experimental autoimmune encephalomyelitis was markedly reduced in IL-9R-deficient mice compared to wild-type cohorts, as CD4^+^ T cells and macrophages from these mice secreted less IL-17 and IL-6, respectively (148). Importantly, IL-9 also plays roles in regulating transplant tolerance, promoting anti-parasitic immunity, exacerbating allergy and autoimmunity (149). The role of IL-9 in tumor immunity has been controversial, both promoting antitumor immunity and enhancing transformation and tumor growth. It has been reported that IL-9 overexpression promotes cell proliferation, metastasis and survival of pancreatic cancer and lymphomas (150–152). However, the adoptive transfer of antitumor Th9 or Tc9 cells regress melanoma in mice through IL-9-dependent mechanisms and are highly cytolytic, hyperproliferative, and persistent post transfer into animals (153–157).

### IL-15

As depicted in Figure 1, IL-2 and IL-15 share common receptor subunits, IL-2Rβ and the γc, only differing between the unique α subunits (53). IL-15Rα is expressed on activated monocytes and dendritic cells and because of IL-15Rα's high affinity for IL-15, IL-15 can be trans-presented to IL-2Rβ and the γc on NK and CD8^+^ T cells, unlike IL-2 which is primarily cis-presented (53, 158). Also, in contrast to IL-2, IL-15 is primarily produced by innate immune cells (including dendritic cells, macrophages and monocytes) (159–162). IL-15 and IL-15R signaling are important for the development and homeostasis of NK cells and CD8^+^ T cells though the up-regulation of anti-apoptotic markers Mcl-1 and Bcl-2 while inhibiting AICD (163–174). This discovery became clear in studies using IL-15- and IL-15R-deficient mouse models, which have impaired NK cell and CD8^+^ T memory cell development and compromised lymph node homeostasis (164, 175). IL-15, unlike IL-2, preferentially expands CD8^+^ T cell memory and NK cells in the presence of Treg cells while promoting resistance to Treg suppression (176, 177).

### IL-21

IL-21 has been reported to improve antitumor T cell immunity but has also been identified as a potent mediator of autoimmunity (178). IL-21 is primarily produced by activated CD4^+^ T cells, particularly Th17 and Tfh but can also be produced by NKT cells (179–181). As shown in Figure 1, the IL-21 receptor (IL-21R) is comprised of the γc and IL-21Rα (182, 183). Receptor expression is low on resting T cells but is upregulated upon TCR activation or IL-21 stimulation (183–185). Both adaptive and innate immune cells are influenced by IL-21 as T, B, NKs, macrophages and DCs all express the IL-21R (179, 181, 183, 184, 186). IL-21 promotes the proliferation, survival and differentiation of Th17 and Tfh subsets while enhancing the function of cytotoxic CD8^+^ T cells (187–197). Additionally, IL-21 blunts Treg expansion by suppressing Foxp3 expression and favors the enrichment of antigen-stimulated CD8^+^ T cells (198). Th17 and Th2 immune responses are impaired while Tregs are increased in IL-21- and IL-21R-deficient mice (190, 191, 199, 200).

Collectively, γc cytokines play a major role influencing the development, differentiation, and survival of innate and adaptive immune cells. For cancer treatment, γc cytokines have been used systemically as monotherapies to harness endogenous immune responses, or in combination with ACT to improve antitumor efficacy. The presence of γc cytokines at various points in the T cell development including priming, *ex vivo* expansion, or post adoptive transfer can influence the function of tumor-specific T cells. As both IL-4 and IL-9 have not been thoroughly explored for ACT and have controversial roles in both promoting tumorigenesis and mediating antitumor immunity, we will focus the rest of our discussion on the clinical uses of IL-2, IL-7, IL-15, and IL-21 for immunotherapy, and their potential to improve patient responses to T-cell based therapies.

## Clinical Uses of IL-2, IL-7, IL-15, and IL-21 in Cancer Immunotherapy

### Interleukin-2: T Cell Proliferation at the Cost of Treg Expansion

Currently, IL-2 is the only γc cytokine to be FDA-approved to treat patients with cancer. In anti-cancer therapies, this cytokine is commonly administered to patients to augment the engraftment and function of adoptively transferred T cells. For treatment of several autoimmune disorders such as type 1 diabetes, HCV-induced vasculitis and graft vs. host disease (GVHD), IL-2 is administered at low doses and has been beneficial for patients because it targets the constitutive expression of the high affinity IL-2R leading to selective proliferation of Tregs (201–204). Conversely, effector T cells do not readily express the high affinity IL-2R. High dose IL-2 is administered to cancer patients to support the proliferation and function of cytotoxic T lymphocytes (CTLs) (205, 206). In fact, since the 1980s high dose IL-2 has been used to treat patients with renal cell carcinoma and metastatic melanoma (207–210). Standard treatment protocols involve the administration of 720,000 IU IL-2/kg every 8 h for up to 14 consecutive doses. Using high-dose IL-2 for patients with renal cell carcinoma, 14% of patients (255 patients total) had an objective response, while 12 patients experienced a complete response (209). Similar efficacy was observed with high-dose IL-2 treatment for metastatic melanoma, where 16% of patients (270 patients total) had an objective response with 17 patients having a complete response and 26 patients experiencing a partial response (210). High dose IL-2 treatment was FDA-approved for renal cell carcinoma in 1992 and for metastatic melanoma in 1998 (211, 212). However, due to toxicities associated with this therapy such as hypotension, capillary leak syndrome, cardiac toxicity, and renal failure, many cancer centers stopped using this therapy to treat patients (213–215). Today, IL-2 is mainly used to expand TILs or CARs *ex vivo* for ACT and is administered to the patient to support donor cell expansion post-transfer.

**Figure 2:**
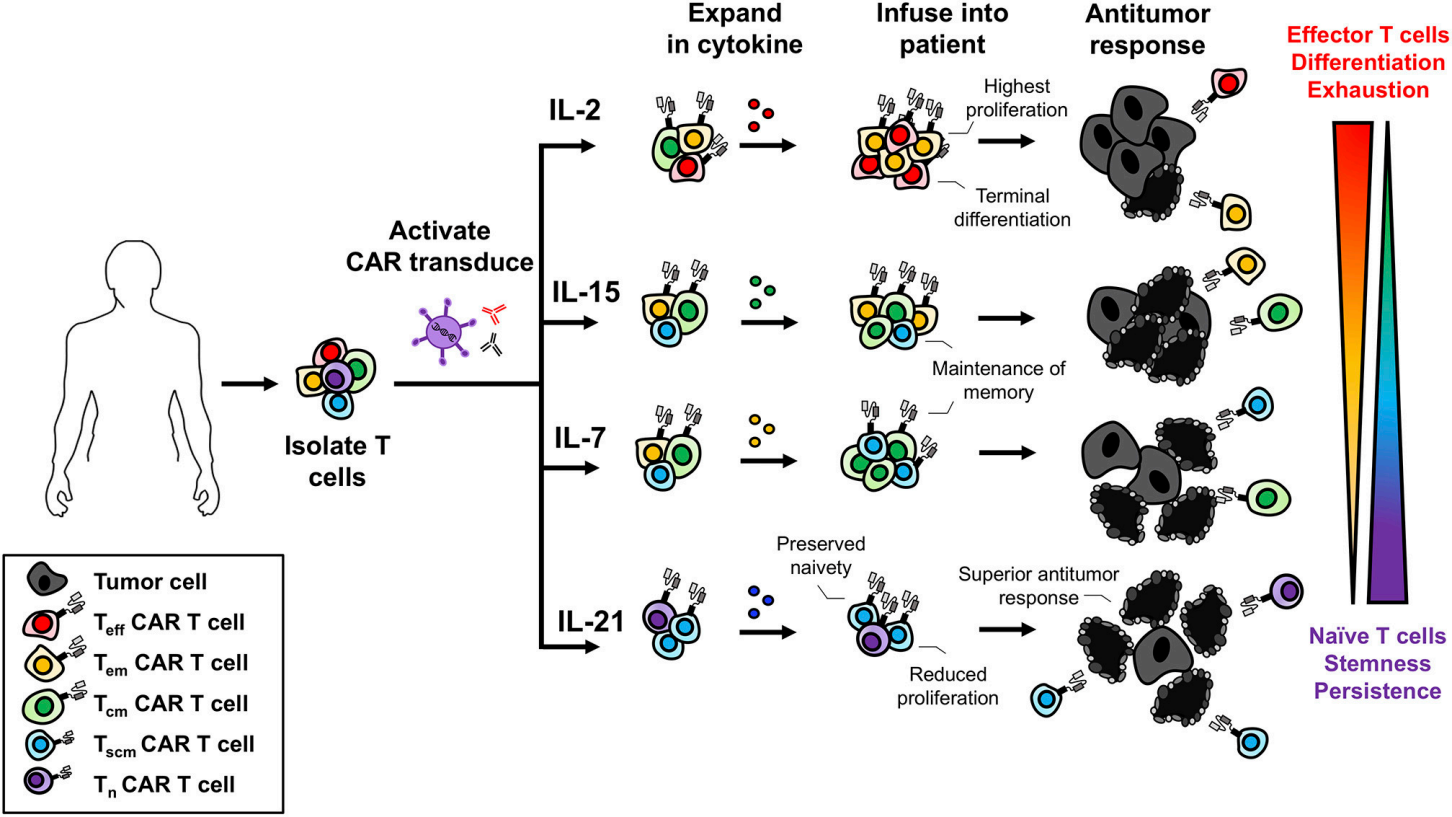
Use of γc cytokines for *ex vivo* T cell expansion generates T cells with variable memory phenotypes. γc cytokines promote different biological programs that influence the differentiation of T cells. While IL-2 promotes robust proliferation it also promotes terminal effector differentiation. IL-7 and IL-15 maintain the homeostasis and survival of memory T cells and treatment *ex vivo* promotes a Tscm/Tcm phenotype. IL-21 slows T cell expansion but prevents differentiation and maintains a naïve-like T cell phenotype. With respect to antitumor immunity, less differentiated cell products are more therapeutic leading to the understanding that IL-2 is not the best option for *ex vivo* expansion.

As IL-2 promotes the differentiation of naive CD8^+^ T cells to full effectors and generates Tregs in the ACT products (Figure 2), immunologists have focused on preferentially targeting IL-2 to effector T cells. One promising way to target IL-2 to effectors has been by complexing this cytokine with anti-IL-2 antibodies. This IL-2 complex uniquely presents IL-2 to the intermediate but not high affinity IL-2Rs thereby reducing Treg expansion (216–219). The importance of targeting IL-2 to transferred T cells has also shown promise in the field of cancer immunotherapy. For example, Rubinstein and colleagues discovered that IL-2Rα on transferred T cells sustained signaling by promoting recycling of endocytosed IL-2 back to the cell surface (220). This recycling mechanism raised the possibility of engineering TILs or CARs to express IL-2Rα to improve IL-2-based therapies (220). Furthermore, other groups have recently discovered novel ways to specifically target transferred T cells with IL-2. In fact, Sockolosky et al. engineered a synthetic IL-2 and IL-2R (distinct from native IL-2 and the IL-2R) and expressed them on transferred T cells. The synthetic IL-2R did not interact with native IL-2, could mediate IL-2R signaling, thereby leading to the selective proliferation of CTLs and regression of melanoma in mice (221).

TIL therapies require expansion of ample numbers of lymphocytes from the suppressive tumor microenvironment. *Ex vivo*, patient tumor samples are treated with high dose IL-2 to preferentially expand TIL. These TIL are then rapidly expanded in the presence of anti-CD3, IL-2 (6000IU/mL) and irradiated feeder cells for several weeks in order to propagate them to the billions (222). After expansion, TIL are infused into the patient who has been preconditioned with a non-myeloablative preparative regimen (2, 212, 223). Upon transfer, IL-2 is administered to patients to promote the expansion of donor TILs *in vivo* because these cells have increased IL-2Rα as expression is positively regulated by TCR and IL-2R signaling (43). In a preclinical model using Epstein Barr Virus positive tumors, EBV-specific CTLs were engineered to express IL-2 or IL-15. Transgenic expression of IL-2 or IL-15 increased T cell expansion *in vitro* and *in vivo* ultimately enhancing their *in vivo* efficacy (224). Treating melanoma patients with *ex vivo* expanded TIL and high dose IL-2 (720,000 IU/kg) led to complete remission in 20 of 93 patients and some patients experienced long-term remission (225). Transducing melanoma TIL to continually secrete IL-2 bypassed the need for exogenous administration of IL-2 to the patient. These modified cells survived in the patient but surprisingly did not improve clinical outcomes compared to TIL administered with exogenous IL-2 (226, 227).

Similar to TIL therapies, IL-2 promotes the proliferation of CAR T cells. Yet this cytokine also drives their differentiation into terminal effector phenotypes. In pancreatic ductal adenocarcinoma xenograft models, treatment of mesothelin-specific CAR T cells with a TNFα and IL-2-secreating adenovirus increased their activation, proliferation and antitumor response in mice (228). IL-2 also increases resistance of CD28-CD3ζ CARs *in vitro* to TGFβ-mediated suppression compared to 4-1BB-CD3ζ CARs. CD28 costimulation activates Lck, which promotes IL-2 production and if Lck is nonfunctional, CAR T cells have impaired antitumor activity (229). It has also been reported that CAR T cells expanded with IL-2 (100 IU/mL) for 3 days, compared to 10 days, generated lymphocytes with an increased proportion of “younger” memory-like cells (230, 231). With longer culture time and increased differentiation, CARs mediated slightly reduced anti-leukemia immunity in mice (230). Ablation of IL-2Rα on CAR T cells did not improve their function but did decrease their expansion capabilities *in vitro* (230). While IL-2 promotes the differentiation of naïve T cells to an effector phenotype, IL-2Rβ signaling has been clearly shown to improve the function of CAR T cells. In a recent study conducted by Kagoya et al. CAR T cells were engineered to express a truncated IL-2Rβ domain (232). This truncated domain increased STAT3 and STAT5 signaling and improved their expansion *in vivo*. When these cells were transferred into mice bearing leukemia or melanoma, they had improved survival and regressed hematological and solid tumors more effectively compared to their traditional CAR cohorts (232). To circumvent the negative attributes of IL-2, investigators have also been turning their focus to other γc cytokines including IL-7, IL-15, or IL-21 which may prove to be better candidates to improve methodology for ACT therapy.

### Interleukin-7: Naïve and Memory Cell Proliferation Without Treg Expansion

Similar to IL-2, IL-7 promotes the proliferation of naïve and memory T cells. Thus, IL-7 is a promising cytokine for cancer immunotherapy. The benefit of IL-7 for ACT was first shown in preclinical models treating CTL's *in vitro* with either IL-7 or IL-2. When transferred into mice, IL-7-treated CTLs controlled metastatic disease to the same extent as those treated with IL-2 (233). In clinical trials using recombinant human IL-7 (rhIL-7) as a monotherapy, IL-7 was shown to be well tolerated by patients with advanced malignancies (234, 235). Rosenberg and colleagues treated a cohort of 12 patients (11 metastatic melanoma and 1 metastatic sarcoma) with 8 doses of IL-7 and found dose-dependent increases in CD4^+^ and CD8^+^ T cells with a decrease in Tregs (234). Following this work, Sportes and colleagues conducted an IL-7 dose-escalation study on 16 patients with non-hematologic non-lymphoid cancers and found similar results with increased CD8^+^ T cells and decreased Tregs (235). TCR-repertoire analysis of T cells indicated a more diverse repertoire, signifying IL-7's role in promoting a broader immune response and the selective expansion of naïve T cells (235). Culturing naïve T cells from healthy donors with IL-7, expands T stem cell memory (Tscm) cells to a greater extent than IL-2 treatment (236). These Tscm were defined as CD62L^+^ CCR7^+^ CD45RA^+^ CD45RO^+^ IL-7Rα^+^ CD95^+^ and were shown to have increased expansion as well as a high capacity for self-renewal (236). IL-7 preferentially expanded naïve T cells to a Tscm phenotype compared to Tcm and Tem, likely because naïve cells express more IL-7R, as portrayed in Figure 2. As Tscm have been reported by several groups to mediate potent memory responses to tumors, it has become increasingly clear that IL-7 has promise in the clinical setting (237–240).

IL-7 has been used in CAR T cell therapy, often in combination with other cytokines, during the *in vitro* expansion phase. For example, CAR T cells expanded in the presence of IL-7, IL-4 and IL-21 were found to express less inhibitory receptors compared to IL-2-expanded cells. These cells also had an increased Tscm/Tcm phenotype and co-expressed CD27 and CD28 (241). In the presence of IL-7 and IL-15, CAR T cells possess a naïve/Tscm phenotype with improved proliferation upon antigenic-rechallenge compared to IL-2-treated CARs (242, 243). IL-7/IL-15 expanded CAR T cells have increased *in vivo* persistence, leading to improved antitumor immunity (244). MUC-1-specific CAR T cells have been engineered with a switch receptor containing an IL-4 ectodomain and an IL-7 endodomain to counter the IL-4-rich tumor microenvironment (245). By converting an IL-4 signal into an IL-7 signal, these T cells expanded robustly and mediated potent antitumor immunity in mice bearing breast tumors (245). Anti-CD20 CAR T cells engineered to express CCL19 and IL-7 migrate and expand to a greater extent than conventional CARs and led to complete remission of mastocytoma and Lewis lung carcinoma in mice (246). Additionally, IL-7 was critical to this response, as anti-IL-7Rα administration diminished the therapeutic benefit of these cells (246). Shum and colleagues engineered a GD-2-specific CAR T cell with constitutive IL-7R signaling, CD34 ectodomain and an IL-7Rα endodomain, leading to constitutive STAT5 activation (247). These CARs were able to undergo multiple rounds of expansion and mediate a robust response against glioblastoma and metastatic neuroblastoma tumors (247). These CAR constructs highlight the beneficial role of IL-7 and IL-7R signaling in improving the antitumor functions of T cells for ACT.

### Interleukin-15: CD8^+^ Memory T Cell Expansion With Some NK Cell Assistance

IL-15R signaling is promising for T cell-based cancer immunotherapies. This pathway selectively induces the expansion and function of CD8^+^ memory and NK cells (163–168). For ACT, IL-15 has been used to enhance the activity of TIL and CAR T cells *ex vivo*. Moreover, IL-15 has also been complexed with IL-15Rα and this novel agent has been used as an immunotherapy in cancer patients *in vivo* (248). When cultured *in vitro*, T cells expanded with IL-15, rather than IL-2, are predominately a Tcm phenotype with very few Tem (249, 250). Conversely, IL-2-expanded cells are mostly effectors (Figure 2) (249). In turn, IL-15 generates a cellular product that mediates improved antitumor immunity, as IL-15-propogated Tcm have an improved engraftment potential and migratory capacity compared to IL-2-expanded cells (250, 251). Administration of recombinant IL-15 can promote immunity through the expansion of endogenous CD8^+^ T cells and NK cells (252, 253). In combination with checkpoint inhibition, IL-15 improved CD8^+^ T cell function marked by increased IFNγ production and mice treated with the combination had improved control of metastatic disease (253). Additionally, IL-15 is able to reverse tumor-tolerant CD8^+^ T cells, when IL-2 and IL-7 were unable to, restoring antigen responsiveness and leading to tumor clearance (254–256). In the first clinical trial using recombinant human IL-15 (rhIL-15) to treat 18 patients with metastatic cancer (11 metastatic melanoma and 7 renal cell carcinoma), rhIL-15 was administered intravenously in a dose escalating study for 12 consecutive days (257). From the 18 patients treated, there was only stable or progressive disease. The dosing regimen led to elevated serum levels of IL-6 and IFNγ along with grade 3 toxicities such as hypotension, lymphopenia and elevated aspartate and alanine aminotransferases at higher doses. However, minutes after administration of rhIL-15, NK, γδ, and CD8^+^ T cells effluxed from the blood and proliferated robustly for many days after administration (257). This study implicates the promising role of IL-15 to selectively target the homeostasis and expansion of NK and CD8^+^ T cells.

Complexing IL-15 with IL-15Rα drastically increases the half-life of this cytokine, maximizing its activity while preferentially presenting IL-15 to cells expressing IL-2Rβ and γc (258, 259). For example, ALT-803, an IL-15/IL-15Rα sushi domain complex, mediated improved therapeutic benefit over native IL-15 (260, 261). Administration of ALT-803 in mice led to selective expansion of NK and CD8^+^ T cells with no expansion of Tregs, increased production of IFNγ, TNFα, and IL-10, and reduced metastasis of breast carcinoma, colon carcinomas, and myeloma in mice (260, 261). Therapeutic benefit was mediated by CD8^+^ T cells as their depletion diminished antitumor immunity (260–262). In a phase 1b clinical trial conducted by Wrangle et al. ALT-803 was administered with nivolumab (anti-PD-1) to 21 patients with metastatic non-small cell lung carcinoma (263). ALT-803 could be safely administered to these patients in combination nivolumab. In fact, there were no dose limiting toxicities experienced by patients on this trial (263). Moreover, this therapy dramatically increased the proliferation of NK and CD8^+^ T cells in the blood (263). Although this study was not designed to assess efficacy, the authors reported evidence of the re-induction of antitumor responses in patients who failed to respond to nivolumab therapy alone (263). This study emphasizes the promise of using IL-15/IL-15Rα complex in cancer therapy and also implies that ALT-803 may improve the antitumor activity of TIL or CAR therapies in patients without increased toxic side effects.

In a clinical trial treating 22 patients with advanced stage lymphoma, patients with positive tumor responses and complete remissions had increased IL-15 serum levels (12). Investigators have engineered IL-15-producing CARs to enhance T cell memory development and incorporate NK cell responses for tumor clearance *in vivo*. Anti-leukemia CAR T cells that express IL-15 have increased expansion, viability, and improved antitumor immunity compared to conventional CAR T cells in lymphoma xenograph models (264). In glioma xenograph models, IL-13Rα2-specific CAR T cells that secrete IL-15 showed increased proliferation, sustained cytokine production and improved survival (265). In this model, tumor relapse was observed due to the expansion of tumor cells that had lost expression of the target antigen. However, in some instances retroviral transduction of IL-15 can transform human primary T cells leading to prolonged cell survival, increased telomerase activity and resistance to apoptosis (266). Membrane-bound IL-15 on CAR T cells mediated similar results, as demonstrated by their increased persistence and immunity against leukemia (238). Thus, IL-15 bolsters NK and CD8^+^ T cell expansion and function, which leads to improved immunity, implicating IL-15 as a beneficial cytokine for ACT (237). In the future, it will be paramount to understand the best way to deliver IL-15 therapy in combination with ACT and CAR T cell therapy.

### Interleukin-21: Preventing T Cell Differentiation to Increase Antitumor Immunity

In a phase 1 clinical trial using recombinant human IL-21 (rhIL-21) in a dose-escalation study with 43 patients (24 melanoma and 19 renal cell carcinoma patients), rhIL-21 was administered consecutively for 5 days for two full cycles. rhIL-21 was safe for patients and mediated antitumor immunity in some individuals, as demonstrated by 1 complete response and 4 partial responses (267). To follow this trial, Davis et al. conducted a phase IIa clinical trial treating 24 patients with metastatic melanoma with 30 μg/kg doses of IL-21 (268). Treatment with IL-21 led to 1 complete response and 1 partial response in this study. Additionally, IL-21 lead to the selective activation of NK and CD8^+^ T cells marked by increased expression of CD25, IFNγ, perforin and granzyme B (268). In a phase II trial with 40 metastatic melanoma patients, most of which had metastasis to the lungs, liver or lymph nodes, were treated with either 30 μg/kg or 50 μg/kg of IL-21 (269). Nine patients experienced partial responses where 16 patients had stable disease. There were 6 patients who experienced some dose-limiting toxicities amongst the treatment groups (267). Collectively, these trials indicate the benefit of IL-21 as a monotherapy and warrant the investigation combining IL-21 with other agents for cancer therapy.

IL-21 augments ACT therapy by preserving T cells in a less differentiated state *ex vivo* (88, 237, 250, 270, 271) (see Figure 2). While IL-2 drives robust proliferation and differentiation of CD8^+^ T cells, IL-21 enriches CD8^+^ T cells with a “younger” phenotype that express less IL-2Rα, CD44, and Eomes but have reduced expansion compared to those expanded with IL-2 (88). However, when IL-21-stimulated CD8^+^ T cells were transferred into mice, they mediated superior anti-melanoma immunity compared to T cells treated *in vitro* with IL-2 or IL-15 (88). Additional investigation revealed that IL-21 supported the propagation of lymphocytes that expressed CD62L and secreted IL-2, consistent with Tscm phenotype. Moreover, these cells expressed Tcf1 and Lef7, which are transcription factors critical for the self-renewal of stem cells (88, 272, 273). The benefits of IL-21 have been demonstrated on TILs isolated from ovarian or non-small cell lung carcinoma patients. While IL-2 greatly bolsters TIL expansion, IL-21 is unable to expand TIL alone (274). Importantly, IL-21 does not support the expansion of Treg cells in contrast to IL-2 (274). For human CD8^+^ T cells isolated from the peripheral blood of healthy donors, IL-21 promotes Tscm development *in vitro* leading to improved immunity upon adoptive transfer into mice with melanoma compared to IL-2-stimulated CD8^+^ T cells (275). In addition to using IL-21 for treatment of CD8^+^ T cells *ex vivo*, IL-21 is a potent agent for the expansion of NK cells. Using membrane-bound IL-21 on artificial antigen-presenting cells, NK cells can be expanded to large numbers to elicit graft vs. leukemia responses without inducing GVHD (276, 277). These expanded NK cells had increased cytotoxicity and cytokine production without exhaustion. When combining membrane-bound IL-21 expansion with IL-18, IL-15, and IL-12, NK cells had increased expression of IFNγ and TNFα. These results indicate IL-21 as a potent agent for improving efficacy of T and NK cells for ACT (278).

IL-2 and IL-21 regulate opposite immune programs (88, 279). However, IL-21 is able to synergize with IL-7 and IL-15. For example, IL-15 and IL-21 synergistically promote the expansion CD8^+^ T cells with a Tscm phenotype and have increased persistence when infused into the host (196). Also, cells stimulated with IL-15 and IL-21 mediated enhanced immunity in mice with melanoma compared to T cells expanded in the presence of either IL-15 or IL-21 alone (196). Together these cytokines increase the effector molecules and cytokines produced by T cells *in vitro* (280, 281). Likewise, combining IL-7 and IL-21 promotes the expansion of cells with a Tscm phenotype with high CD28 and CD27 expression (241). The synergy between these two cytokines may be due to IL-21 augmenting IL-7-induced expansion of T cells and by preventing the down regulation of IL-7Rα, all of which lead to increased immunity *in vivo* (282). IL-21/IL-7-treated cells have increased proliferation and production of inflammatory cytokines, directing improved lysis of tumor cells (282). These data support the use of cytokines in combination in next generation clinical trials for patients.

As IL-21 prevents T cell differentiation and preserves their naïve-like phenotype, investigators have used this cytokine to generate “younger” CAR T cells. Interestingly, culturing CD19 CD28-CD3ζ CARs with IL-21 led to CAR^+^ T cell expansion and increased expression of IFNγ and granzyme B (283). Compared to IL-2-treated CARs, IL-21-treated CARs had increased expression of CD45RA, CD62L, CCR7, and CD28. When transferred into mice with leukemia, IL-21-treated CARs had improved tumor control compared to those treated with IL-2 *ex vivo* (283). Moreover, membrane-bound IL-21 on CAR T cells recapitulated the effects of soluble IL-21 in culture (283). To improve the activity of CAR T cells, Sabatino et al. isolated naïve CD8^+^ T cells (CD62L^+^ CD45RA^+^ CCR7^+^) from healthy donors and transduced them with CD19 CD28-CD3ζ CAR constructs. During expansion, cells were cultured in IL-7, IL-21 and TWS119 (glycogen synthase kinase 3β inhibitor), which enriched for Tscm (284). CD19-CAR Tscm had no changes in their transcriptome compared to non-transfected Tscm and were polyfunctional (284). When transferred into mice with leukemia, CD19-CAR Tscm cells were maintained with intraperitoneal injections of IL-15 and displayed improved survival over conventional CD19-CAR T cells (284). This study demonstrates that *in vitro* cooperation of IL-7, IL-21, and TWS119 and the *in vivo* functions of IL-15, lead to improved T cell functionally improving therapeutic outcome *in vivo*.

### TRUCKs: Putting Cytokines to Work in the Tumor Microenvironment

Engineering CAR T cells with inducible or constitutive cytokine secretion reinforces transferred T cell function in the host and manipulates the endogenous immune response within the tumor. T cells redirected for universal cytokine-mediated killing, termed TRUCKs, are such CAR T cells equipped with the expression of IL-2, IL-7, IL-15, or IL-21 (285, 286). In a study conducted by Markley and Sadelain, human CD19-CAR T cells were engineered to constitutively express either IL-2, IL-7, IL-15, or IL-21 (285). Using lymphoma model, constitutive expression of the γc cytokines improved antitumor immunity and animal survival. Even though IL-2- and IL-15-expressing TRUCKs led to the upregulation of effector molecules such as granzyme A, TNFα and IFNγ, TRUCKs that produced IL-7 or IL-21 were most efficacious (285). IL-21-expressing TRUCKs mediated the best overall tumor immunity in mice, demonstrated by their capacity to increase survival. These TRUCKs were found to co-express CD27 and CD28 and were able to persist long-term in the animals (285). IL-7-expressing TRUCKs mediated improved antitumor immunity, while upregulating Bcl-2 expression and promoting improved cell expansion *in vitro* compared to IL-2-expressing TRUCKs. This preclinical study suggests that IL-7 or IL-21 TRUCKs could be efficacious in patients.

Other research has revealed that cytokines not in the γc cytokine family, such as IL-12 and IL-18, could be efficacious in TRUCK constructs. For example, in ovarian carcinoma xenograft models, MUC-16^ecto^-specific second-generation CAR T cells were engineered to secrete IL-12 which led to improved expansion of TRUCKs and a 27-fold increase in IFNγ production compared to non-IL-12-secreting constructs (287). MUC-16^ecto^-specific IL-12-secreting TRUCKs had enhanced immunity compared to non-IL-12-secreting CARs in mice with ovarian cancer, leading to near complete survival (287). IL-18-secreting TRUCKs have similar benefits to IL-12-secreting TRUCKs with enhanced immunity and increased proliferation in both mice and humans (288). However, IL-18 secretion had preferential effects on CD4^+^ TRUCKs and was able to promote significant T cell expansion without costimulatory signaling. IL-18-secretion expanded both CAR^+^ and CAR^−^ T cells in an antigen-independent manner which could be beneficial in cases of epitope spreading but detrimental for autoimmune manifestations (288). As both IL-12 and IL-18 promote immunity and expansion of TRUCKs, these cytokines could be potential candidates to improve therapy for solid tumors. However, as IL-12 and IL-18 upregulate the expression of several inflammatory cytokines, such as IFNγ, and have a historic reputation of toxic side effects, administration could further exacerbate CRS already associated with CAR T cell therapy (289–292). Because of these toxic side effects both preclinically and in patients, the γc cytokines, IL-7, Il-15 and IL-21 might prove to be better options for TRUCK therapies. These cytokines have well documented roles in improving cell products for ACT and as shown by Markley and Sadelain, can improve antitumor immunity of TRUCKs. Additionally, IL-7, IL-15, and IL-21 reinforce the essential T cell functions of proliferation, effector function and memory warranting further investigation. Overall, it is clear that further work must be done to investigate whether the γc cytokine-secreting TRUCKs would be beneficial to overcome the suppressive tumor microenvironment. Findings from future work will be instrumental to apply this therapy to patients with solid tumors, as these constructs have been preclinically shown to be efficacious for blood cancers.

### Conclusion: Ideal Use for γc Cytokines in TIL and CAR T Cell Therapy

**Figure 3:**
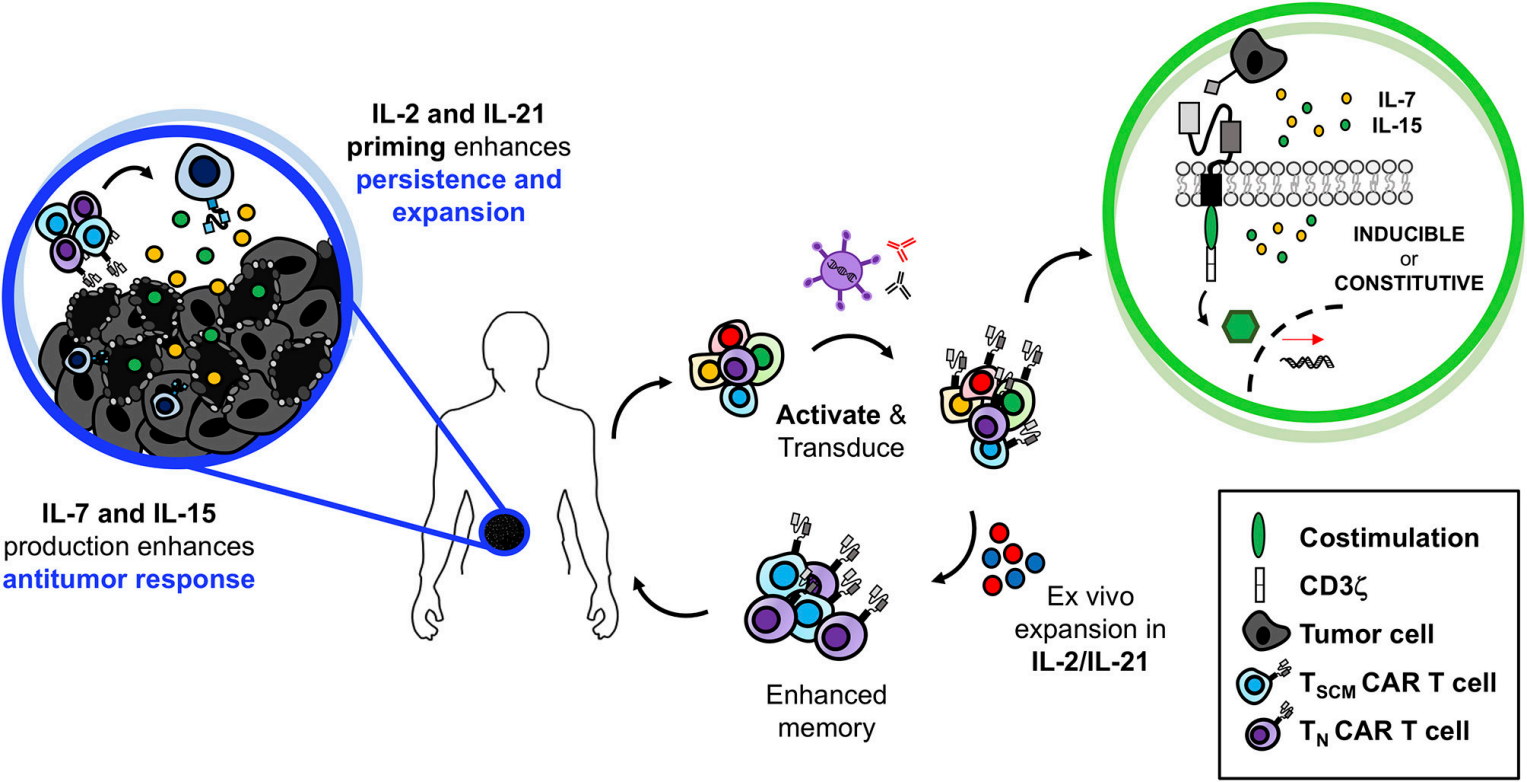
Superior antitumor immunity of IL-2/IL-21-primed CAR T cells producing IL-7 and IL-15 at the tumor site. Generating TRUCKs *ex vivo* in the presence of IL-2 and IL-21 would prevent terminal differentiation, promote enhanced antitumor immunity with robust T cell proliferation. While at the tumor site, secretion of IL-7 and IL-15 would maintain TRUCK proliferation and memory function allowing for robust and persistent antitumor immunity against solid tumors.

In summary, we have discussed how the various γc cytokines play fundamental roles in shaping T lymphocyte biology. We have also highlighted important preclinical work that reveals their potential for immunotherapy via several modalities: (1) infusion as monotherapies or in combination with adoptive T cell transfer therapy, (2) *ex vivo* expansion of TILs and CAR T cells to generate “younger” more agile cell products, and (3) *in vivo* constitutive or inducible production by genetically engineered T cells (TRUCKs) to bolster not only the transferred cells but to enhance immune cells in the oppressive tumor microenvironment. While exploration of TRUCKs has been largely preclinical to date, promising results indicate high potential for successful future clinical translation. Though IL-2 is the only currently FDA-approved γc cytokine, it is possible that this cytokine alone may not be ideal for future trials. As depicted in Figure 3, we envision the ideal application of the γc cytokines for T cell therapy to involve a combinatorial approach. Based on preclinical work, perhaps the ideal way to expand T cells *ex vivo* may require the presence of both IL-21 and IL-2. As published by Hinrichs and team in murine T cells, we propose that IL-21 will effectively prevent the terminal differentiation of T cells while preserving a “younger” phenotype whereas IL-2 will support their expansion to large enough numbers to effectively treat patients (88). Upon administration, we suspect that these IL-21/IL-2-expanded TILs or TRUCKs would be best maintained by engineering them to secrete IL-7 and IL-15, which we hypothesize will further promote their persistence and memory recall responses to prevent tumor relapse in patients. The concept portrayed in Figure 3 is just one of many possible ways to combine γc cytokines to bolster T cell-based therapies. We certainly realize that it is also possible that other cytokine combinations will be important in generating T cells with long-lived responses to aggressive tumors. Future studies are also necessary to turn off inhibitory signals (such as TGFβ and IL-10) that dampen T cell responsiveness (293, 294). Regardless, it has become increasingly clear that the γc cytokine family represents a group of cytokines that support the fundamental attributes T cells and understanding how to exploit these cytokines for therapeutic use is critical for next generation cancer clinical trials involving vaccines, checkpoint inhibitors and ACT therapy.

## Author Contributions

CD and CP conceptualized, wrote and edited the manuscript. CD and HK conceptualized and created the figures. HK, AS, MW, GR, DA, EB, ZL, and MR critically reviewed and provided feedback for this manuscript.

### Conflict of Interest Statement

The authors declare that the research was conducted in the absence of any commercial or financial relationships that could be construed as a potential conflict of interest.
